# Human nail stem cells are retained but hypofunctional during aging

**DOI:** 10.1007/s10735-018-9769-0

**Published:** 2018-04-09

**Authors:** Jia Shi, Zhengtao Lv, Mingbo Nie, Weiwei Lu, Changyu Liu, Yong Tian, Long Li, Guoxiang Zhang, Ranyue Ren, Ziyang Zhang, Hao Kang

**Affiliations:** Department of Orthopedics, Tongji Hospital, Tongji Medical College, Huazhong University of Science and Technology, 1095 Jiefang Avenue, Qiaokou District, Wuhan, 430030 China

**Keywords:** Human nail, Stem cell, Aging, Regeneration

## Abstract

The nail is a continuous skin appendage. Cells located around the nails, which display coordinated homeostatic dynamics and release a flow of stem cells in response to regeneration, have been identified in mice. However, very few studies regarding human nail stem cells exist in the literature. Using specimens isolated from humans, we detected an unreported population of cells within the basal layer of postnatal human nail proximal folds (NPFs) and the nail matrix around the nail root. These cells were multi-expressing and expressed stem cell markers, such as keratin 15 (K15), keratin 14 (K14), keratin 19 (K19), CD29, CD34, and leucine-rich repeat-containing G protein-coupled receptor 6 (Lgr6). These cells were very similar to mouse nail stem cells in terms of cell marker expression and their location within the nail. We also found that the putative nail stem cells maintained their abundance with advancing age, but cell proliferation and nail growth rate were decreased on comparison of young and aged specimens. To summarize, we found a putative population of stem cells in postnatal human nails located at NPFs and the nail matrix. These cells may have potential for cell differentiation and be capable of responding to injury, and were retained, but may be hypofunctional during aging.

## Introduction

The nail is the largest and most complex appendage of the skin in the human body. Skin, constituting the largest organ in our body, functions to defend against external threats, excrete waste from the body, and maintain body temperature (Johansen 2017). Skin and its appendages are in a process of permanent regeneration. Epidermal resident stem cells are found in the outermost layer of mammalian skin. These stem cells are responsible for continuous self-renewal, which sustains tissue homeostasis. There is a point in skin turnover where epidermal cells are found in the basal cell layer, forming epidermal proliferative units (Mackenzie 1970, 1997). Li et al. isolated and purified epidermal stem cells from neonatal foreskin through enzymatic digestion and identified specific epidermal stem cell markers (Jones and Watt 1993; Li et al. 1998). For skin to function, all components, including hair, sweat glands, sebaceous glands, and nails, must contribute. Several previous studies have evaluated and identified different types of skin stem cells (Cotsarelis 2006; Danner et al. 2012; Leung et al. 2013; Lyle et al. 1998; Trempus et al. 2003; Zhu et al. 2014). One stem cell type is that of hair follicle stem cells; they reside in bulge regions, are multi-potent (Oshima et al. 2001), and can differentiate into non-epithelial cells, such as neurons and adipocytes (Toma et al. 2001). Sweat gland-derived stem cells are also multi-potent (Egana et al. 2009). However, there has been little previous research on human nail stem cells.

**Fig. 1:**
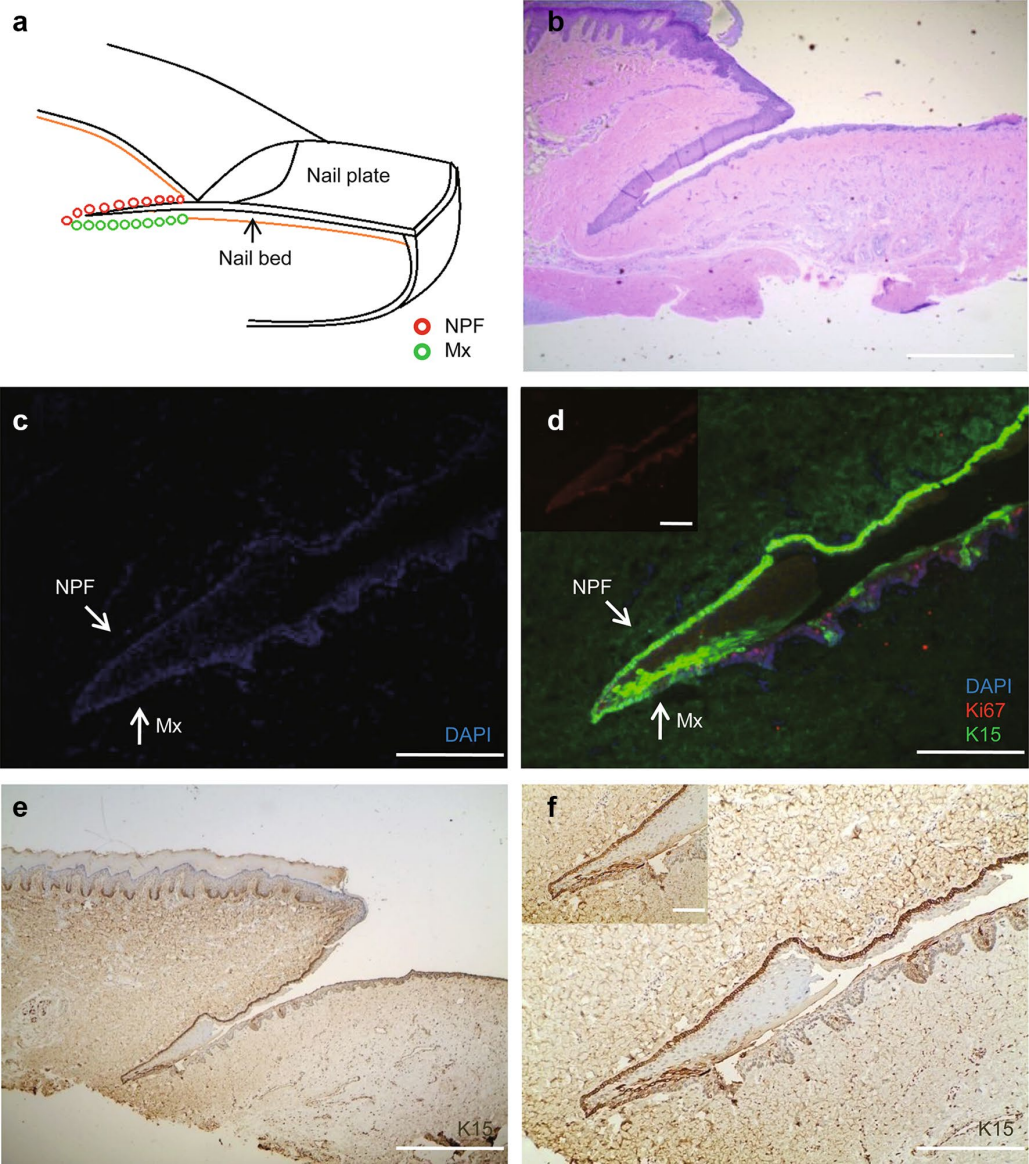
Localisation of stem cells in human nails. Side view, components of the human nail **a** Red circles are cells in nail proximal folds (NPFs); green circles are cells in the nail matrix (Mx). We found K15^+^ label-retaining cells in human nails in both the NPF and Mx. The diagrammatic drawing shows the tangent plane of the slice. **b** H&E staining of human nail side facing showing the nail bed (NB), nail root at the Mx and the NPF junction (scale bar = 1000 μm). **c, d** Immunofluorescence staining for K15 (green) and Ki67 (red); cell nuclei were stained blue by DAPI, image of separately staining of Ki67 in the upper left corner. The positive expression of K15 extended from the PF to the proximal Mx (scale bar = 200 μm). **e** Immumohistochemical staining for K15 (brown) (scale bar = 1000 μm). **f** Magnified photographs of immumohistochemical staining for K15 (brown) (scale bar = 200 μm). (Color figure online)

Human nails are located in the dorsal region of the fingertip and have a protective function (Haneke 2015). Nails begin to form during the ninth week of the embryo’s life and develop a visible nail plate after 5 weeks (Haneke 2015). The nail itself belongs to differentiated tissue (Zaias 1963). A nail unit consists of four components: the nail matrix, nail bed, nail plate, and nail fold (Haneke 2014, 2015) (Fig. 1a). The nail fold is the area of the epithelial fold close to the proximal nail bed, and the NPFs and nail matrix are locations where previous studies have identified stem cells in mice (Lehoczky and Tabin 2015; Leung et al. 2014; Nakamura and Ishikawa 2008). However, to date, there has been little research into postnatal human nail stem cells. Stem cells, which differentiate and contribute to the formation of the nail structure and peri-nail epidermis, have previously been found around the nails in rodents (Lehoczky and Tabin 2015; Leung et al. 2014). For example, Leung et al. found bifunctional stem cells around the nails in mice (Leung et al. 2014). If the same or analogous cells are found in human nails, we may be closer to realising the regeneration of much larger areas of limbs and even the regrowth of whole limbs and other non-regenerating tissues. Previous studies have found that the digit tip blastema consists of different species of progenitor cells (Rinkevich et al. 2011). In human fingertips, only the nail can regenerate after amputation; indeed, it is necessary for the regeneration of the fingertip (Neufeld and Zhao 1995). Takeo et al. 2013 showed that nails contribute to digit tip regeneration at the molecular level. Meanwhile, through rigorous measurements and calculation, we observed that young and aged people have different nail growth rates. However, whether this is related to nail stem cells remains to be confirmed.

In this study, we searched for putative stem cells in human nails to identify their specific locations and to determine any difference in the number and/or function of those cells and their locations during aging. This study was conducted in light of recent discoveries that stem cells around mouse nails were required for digit tip regeneration (Lehoczky and Tabin 2015; Leung et al. 2014). Therefore, identifying human nail stem cells and their regenerative potential may help provide new therapies for patients with defects of the digits, as well as amputees.

## Materials and methods

### Specimens and slice production

All samples used in this study were obtained postnatally from humans. Samples were collected from September 2015 to June 2017. In total, 61 participants were recruited initially, with 58 finally completing the protocol (Table 1). All participants gave written informed consent, and the study was approved by the Ethics Committee at Huazhong University of Science and Technology. We first collected distal finger segments from the study participants, most of whom were children with polydactyly, adults with trauma leading to amputation, or patients with other medical conditions requiring amputation. Due to the slice limitation for the tissues, we removed the nail plate and distal phalanx, leaving only the nail bed and the surrounding soft tissues for paraffin and frozen sections.

**Table 1:** Characteristics of participants

Characteristics	Participants of slice specimens (n = 58)	Participants of nail grow rate (n = 64)
	n (%)	n (%)
Female	22 (37.9)	46 (71.9)
Age group (years)		
1–3	34 (58.6)	34 (53.1)
60–70	24 (41.4)	30 (46.9)
Body mass index (kg/m^2^)		
< 18.5	3 (5.2)	3 (4.7)
18.5–24	49 (84.5)	54 (84.4)
24–28	6 (10.3)	7 (10.9)
≥ 30	0 (0)	0 (0)
Dominant right hand	52 (89.7)	59 (92.2)
Any onychophagia	0 (0)	0 (0)
Medication used in past year	0 (0)	0 (0)
Nail polished	0 (0)	0 (0)
Any chronic disease	3 (5.2)	0 (0)
Any family history of chronic disease	8 (13.8)	12 (18.8)

### Immunohistochemistry and immunofluorescence staining

For all stains, specimens were fixed in 4% [vol/vol in phosphate-buffered saline (PBS)] paraformaldehyde. Tissue sections were stained with hematoxylin and eosin (H&E) for visualisation. For immunofluorescence staining, the tissue was cut into 4-μm cryosections, and the putative nail stem cells were plated onto a 96-well plate and incubated with the following primary antibodies: K14 (1:200; Abcam, Cambridge, UK, ab7800), K15 (1:200; Thermo Fisher Scientific, Waltham, MA, USA, MA5-11344), K15 (1:100; Proteintech Group, 10137-1-AP), K19 (1:200; Abcam, ab52625), CD34 (1:100; BOSTER, Wuhan, China, BA0532), CD29 (1:200; Abcam, ab52971), Lgr6 (1:100; Abcam, ab126747), and Ki67 (1:100; BOSTER, PB0065). The cells were then incubated with the following secondary antibodies: goat anti-rabbit FITC 1:100 (BOSTER, BA1090) and goat anti-mouse TRITC 1:100 (BOSTER, BA1089). DAPI (Thermo Fisher Scientific, D3571) staining was used to display the nuclei.For immunohistochemistry staining, the tissue was cut into 3-μm paraffin sections. Paraffin sections of human nail samples were analysed using the IHC kit (BOSTER, SA1022).

### Isolation of nail stem cells and clonogenicity assays

Human nail stem cells were isolated according to the method previously described by Leung et al. (2014). Briefly, the fingers were collected and the epidermis was carefully cut open at the proximal fold to reveal the nail stem cells. Next, strips of nail stem cells were individually isolated through microdissection and collected in PBS. Next, the isolated strips were digested with 0.25% trypsin–EDTA overnight at 4 °C with shaking. Single cells were filtered through a 40-μm cell strainer and cultured in Defined Keratinocyte-SFM (DKSFM, Thermo Fisher Scientific, 10744019). The culture medium was changed every 3 days. Next, cells were plated onto a 24-well plate, and cell growth was examined and counted daily. For colony growth, equal initial seeding numbers of K15^+^ cells were plated in 12-well plate with 0.3 mM calcium and 15% (vol/vol) serum (Nowak and Fuchs 2009), in triplicate. After 2 weeks in culture, cells were fixed in 10% formalin and stained with crystal violet. Colonies numbers were counted and measured by using Image J.

### Quantification of cell metabolic activity

A Cell Counting Kit-8 (CCK8) (BOSTER, AR1160) was used to test the proliferative abilities of the cells. Nail stem cells were plated onto a 96-well plate (1000/well) at days 3 and 7 after seeding. Medium was removed and CCK reagent (10 µL) was added to each well and incubated for 1–4 h. To quantify metabolic activity, absorbance was measured at 450 nm and wells without cells were used as a negative control.

### Flow cytometry

Cells were digested with trypsin–EDTA, washed twice with PBS, centrifuged, resuspended in cold 70% ethanol, and kept at 4 °C overnight. Fixed cells were then washed twice with PBS and incubated with RNase at 37 °C for 30 min (Thermo Fisher Scientific, AM2269) and propidium iodide for 20 min (Thermo Fisher Scientific, P3566) in the dark. Absorbance was measured at 488 nm by flow cytometry (BD, USA, FACSort).

### Measurement of nail growth rate

Study participants were recruited from May 2017 to August 2017. Young participants were 1–2 years old and elderly participants were over 60 years of age. In total, 72 participants were recruited and 64 completed the study (Table 1). Exclusion factors included use of nail polish, onychophagia, use of medication, and chronic disease. All participants gave written informed consent, and the study was approved by the Ethics Committee at Huazhong University of Science and Technology. Participants were provided with a flexible ruler, a nail file, standardised forms for recording the measurements, and a study protocol. Nail length was measured according to Dawbe (Dawber 1970). At baseline, we marked the participants’ fingernails near the proximal nail fold by using a nail file with moderate weight. We recorded the first and last date and the distance in millimeters from the proximal nail fold to the mark at two different time points. Participants were free to clip their nails, and nail clipping of infants was performed by a guardian. The nail growth rate was obtained by taking the difference in recorded distance from the proximal nail fold and dividing by the number of days between the two measurements (Yaemsiri et al. 2010). We compared the left and right nail growth rates and tested whether there were significant differences using paired t tests. We also compared mean nail growth rates and the growth rate of each fingernail in the young and old groups using paired t tests.

### Statistical analysis

Experiments were performed three times with similar results. All data are given as the mean ± 95% confidence intervals. The t test was used to compare the differences between the two groups. Statistical significance was defined as *p* < 0.05.

## Results

### Identification of human nail stem cells ex vivo

To locate and identify human nail stem cells, we used extracorporeal specimens from children with polydactyly and severed fingers from adults with trauma leading to amputation (Fig. 1a). H&E staining revealed the morphology of the human nail root (Fig. 1b). We detected the expression of stem cell markers around the nails and used Ki67 as a cellular marker for proliferation (Fig. 1d). We observed strong K15 expression (Fig. 1c–f), which was also apparent in epidermal, sweat gland and hair follicle stem cells (Bose et al. 2013; Leung et al. 2013; Takeo et al. 2013). Through the comparison of the photographs with multiple magnifications, we could easily found that those K15^+^ cells were located at the basal layer. Specifically, K15^+^ cells are located at both the NPF above the nail plate and proximal to the nail matrix. This result not only differed from Leung et al.’s (2014) reports who only found nail stem cells just at the proximal nail fold, but also other reports that identified nail stem cells only in the nail matrix (Lehoczky and Tabin 2015; Nakamura and Ishikawa 2008; Takeo et al. 2013). To ensure that this result was not a coincidence, we treated 58 human specimens following the same steps and obtained similar results.

### High expression of K14, K15, K19, CD29, CD34, and Lgr6 in human nail stem cells and cell abundance are unaffected during aging

**Fig. 2:**
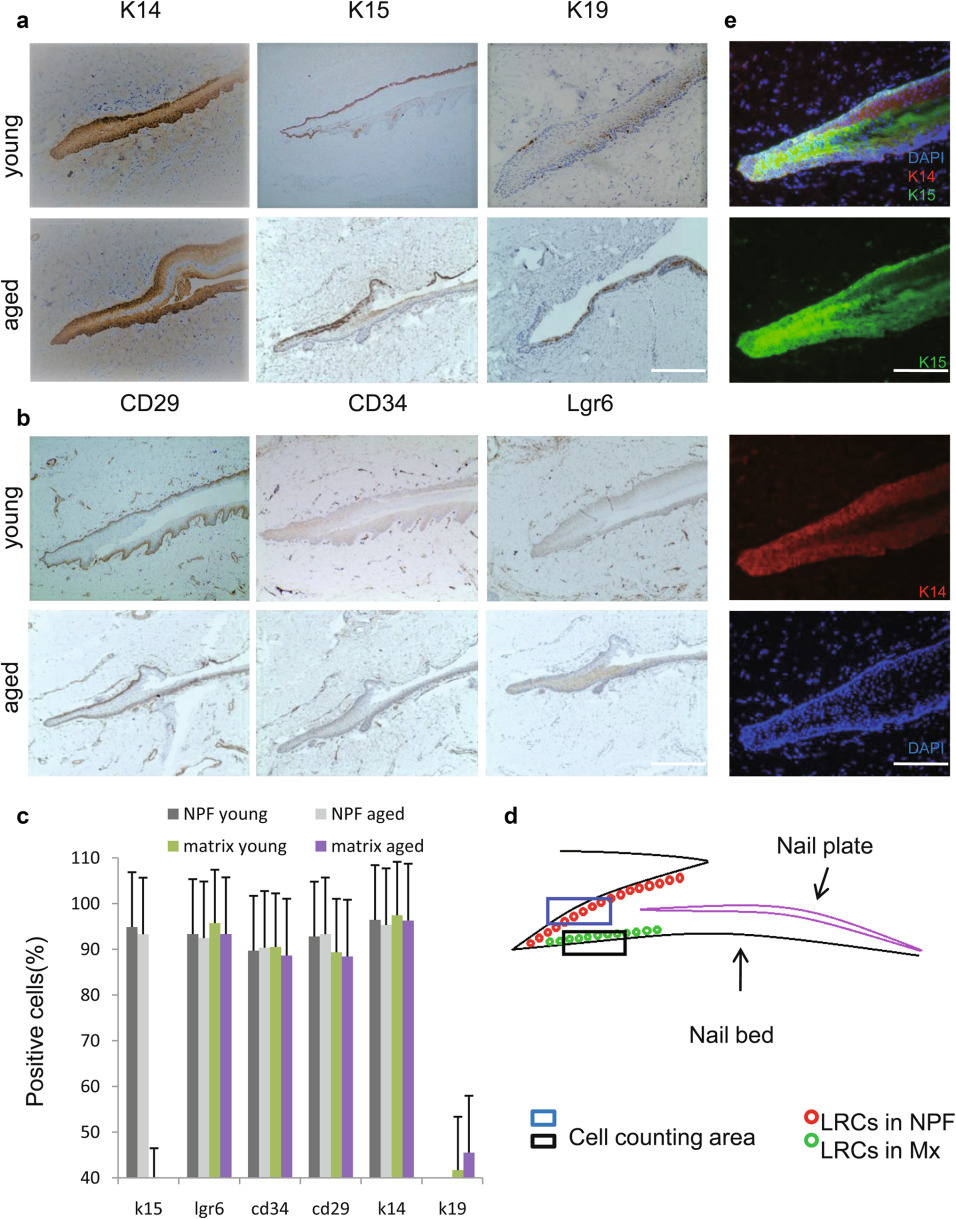
Expression of stem cell markers and the difference between young and aged participants in human nail stem cells. **a** Immunohistochemical staining for K14, K15, and K19 in human nails. These three markers were all expressed in the NPF and Mx in both groups. Compared to K14 and K15, the expression of K19 was relatively weak, but there was no significant difference between the two groups (scale bar = 400 μm). **b** Immunohistochemical staining for CD29, CD34, and Lgr6 in human nails. The markers were expressed in the same area (scale bar = 400 μm). **c** Proportion of positive cells stained for K14, K15, K19, CD29, CD34, and Lgr6 in analysed slices (n = 58, p > 0.05), illustrating no significant difference between the two groups. **d** Diagrammatic drawing of the nail root. The area in the blue and black box was used to determine the percentage of positive cells. **e** Co-immunofluorescence staining for K14 and K15 (scale bar = 400 μm). (Color figure online)

To determine whether stem cells exist in human nails, we investigated multiple biological markers (K14, K15, K19, CD29, CD34, and Lgr6) of skin stem cells in nail-surrounding tissues. The expression of K14, K15, and K19 has been identified in different types of skin stem cells (Bose et al. 2013; Driskell et al. 2015; Garcin et al. 2016; Leung et al. 2013; Morris et al. 2004; Troy and Turksen 1999; Wang et al. 2012; Zgheib et al. 2015; Zhang et al. 2012). CD29 is the most widely accepted of the skin stem cell markers identified (Bose et al. 2013; Watt 1998). CD34^+^ cells are a rich population of skin epithelial stem cells (Hong et al. 2014; Lin et al. 2015; Najafzadeh et al. 2015; Ouji et al. 2015; Zhu et al. 2013). The expression of Lgr6 has been observed in several adult skin stem cells (Barker et al. 2013; Lehoczky and Tabin 2015; Snippert et al. 2010). In human nails, we detected the expression of those six markers in both proximal fold cells and proximal nail matrix cells in all specimens (Fig. 2 a, b), and the expressing regions overlapped, proving the existence of putative human nail stem cells in NPFs and the nail matrix. To analyse the difference between cell numbers in young and aged specimens, we chose the same area of the NPF (Fig. 2d) in each group to count and analyse the cell number (n = 58, p > 0.05, Fig. 2c). We found no significant difference in the proportion of positive cells in each indicator between the young and aged, which indicated that the relative amount of the putative nail stem cells does not change significantly with age. To demonstrate that there is only one putative population of stem cells in human nails, we have performed co-immunofluorescence for K14 and K15 (Fig. 2e). We could see that the cells colored by K14 and K15 were almost completely coincident.

### Immunofluorescence intensity of nail stem cell markers and cell proliferation decrease in an age-dependent and stem cell-independent manner

**Fig. 3:**
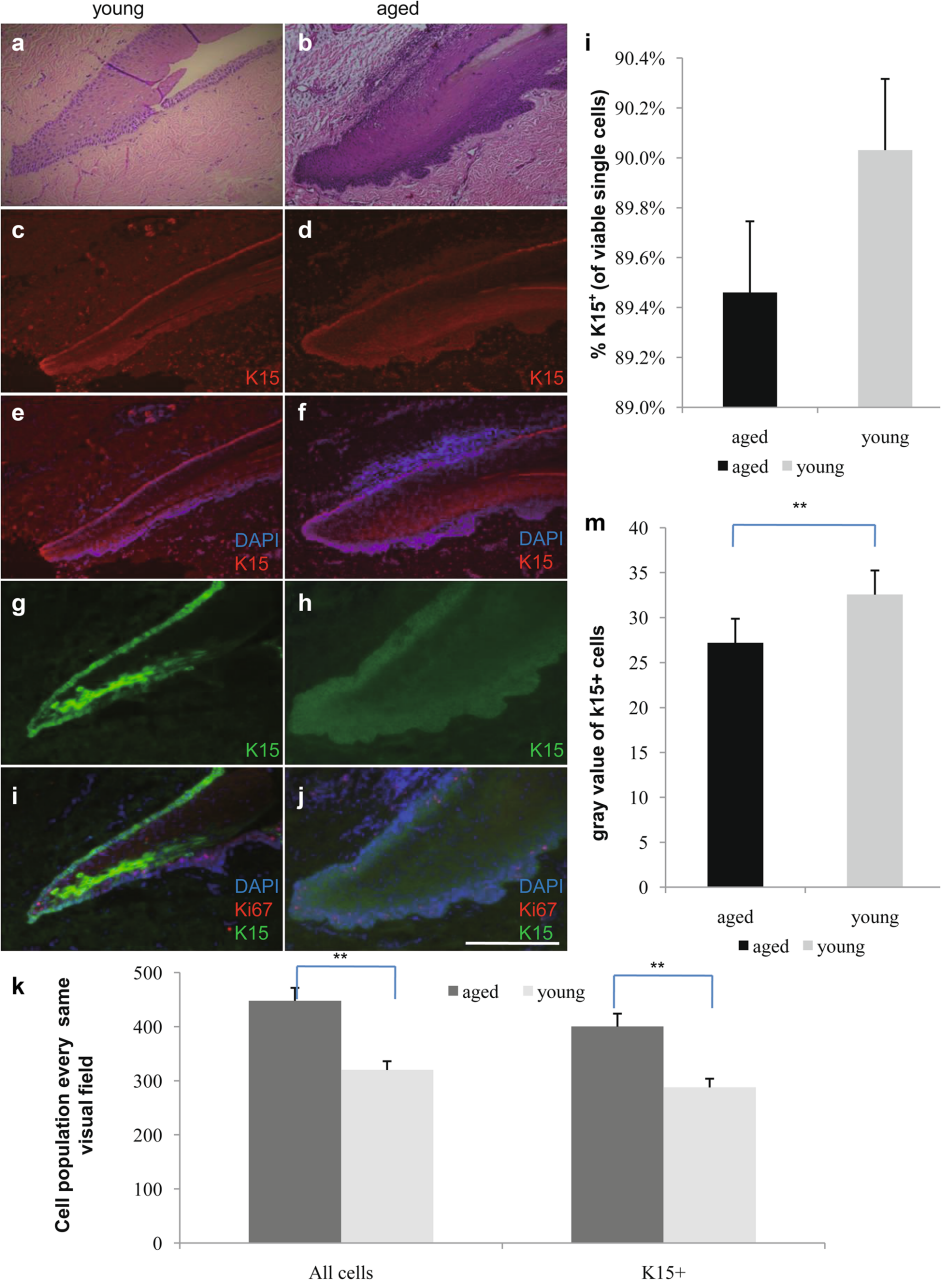
Comparison of cell number and fluorescence intensity between the young and aged groups. **a**–**j** H&E staining of specimens from the young group (**a**) and the aged group (**b**). Immunohistochemical staining for K15 in the two groups: **c, d** K15 (red); **e, f** K15 (red), DAPI (blue); **g, h** K15 (green); **i, j** K15 (green), DAPI (blue), and Ki67 (red) (scale bar = 200 μm). (**k**) Cell counting in the same area in the high-power microscope. The aged group had a higher total cell number and K15-positive cell number as compared to the young group (n = 58, p < 0.01). **l** The proportion of K15-positive cells was around 90% in both groups, with no significant difference (n = 58, p = 0.5), consistent with the immunohistochemical results. **m** Comparison of fluorescence intensity showed that the young group had significantly higher expression of K15 than the aged group (n = 58, p = 0.003), as analysed by Image J. **P < 0.01. (Color figure online)

**Fig. 4:**
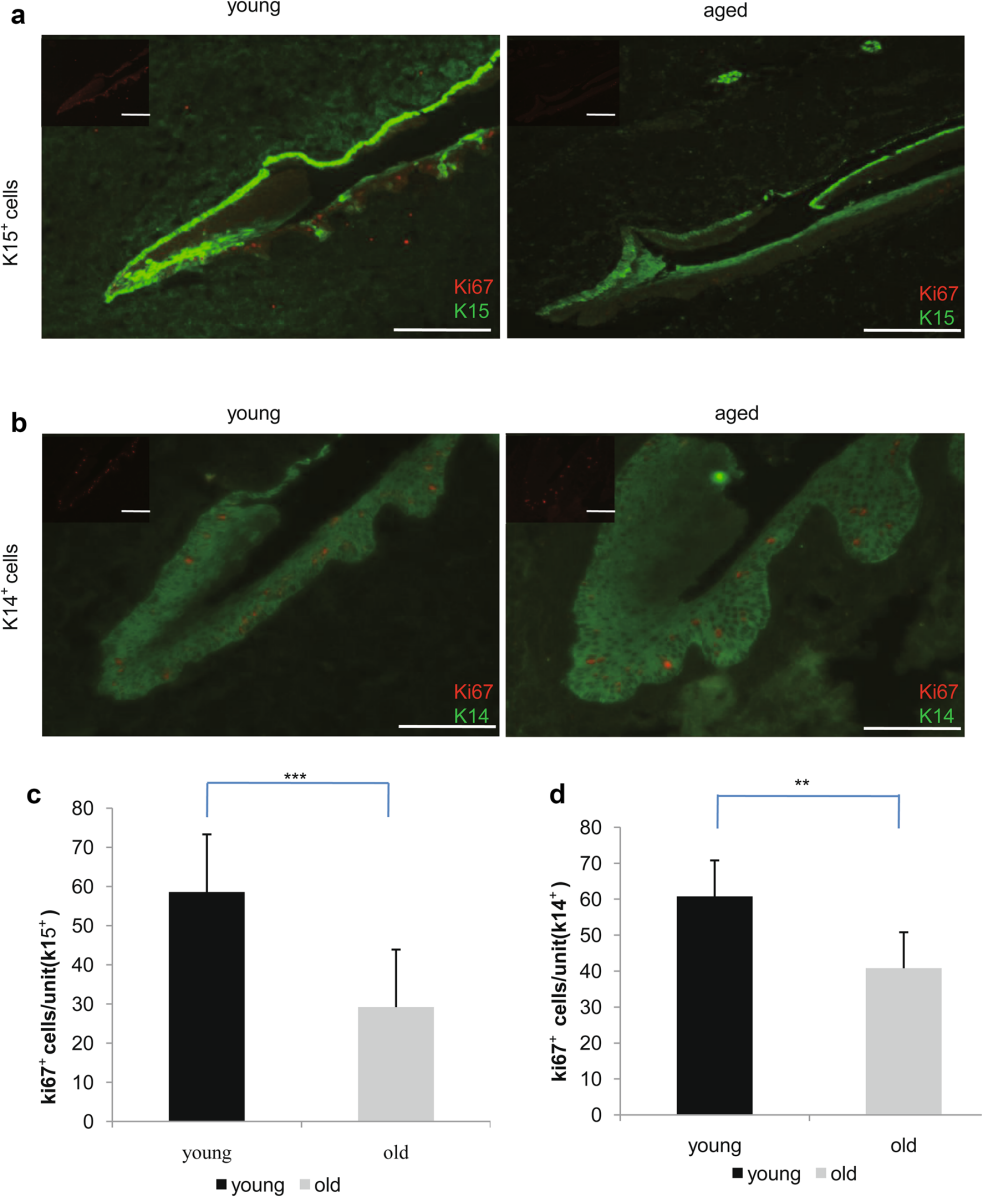
Comparison of cell proliferation between the young and aged groups. **a** Comparison of Ki67-positive cells with K15 positivity in the two groups (scale bar = 200 μm), image of separately staining of Ki67 in the upper left corner. **c** Ki67-positive cell count results; the young group was higher than the aged group (n = 58, p = 0.0005). **b, d** Comparison of Ki67-positive cells with K14 positivity in the two groups and Ki67-positive cell count results (scale bar = 200 μm), image of separately staining of Ki67 in the upper left corner. Similar results were obtained for both K14 and K15 staining (n = 58, p = 0.002). **P < 0.01 ***P < 0.001

**Fig. 5:**
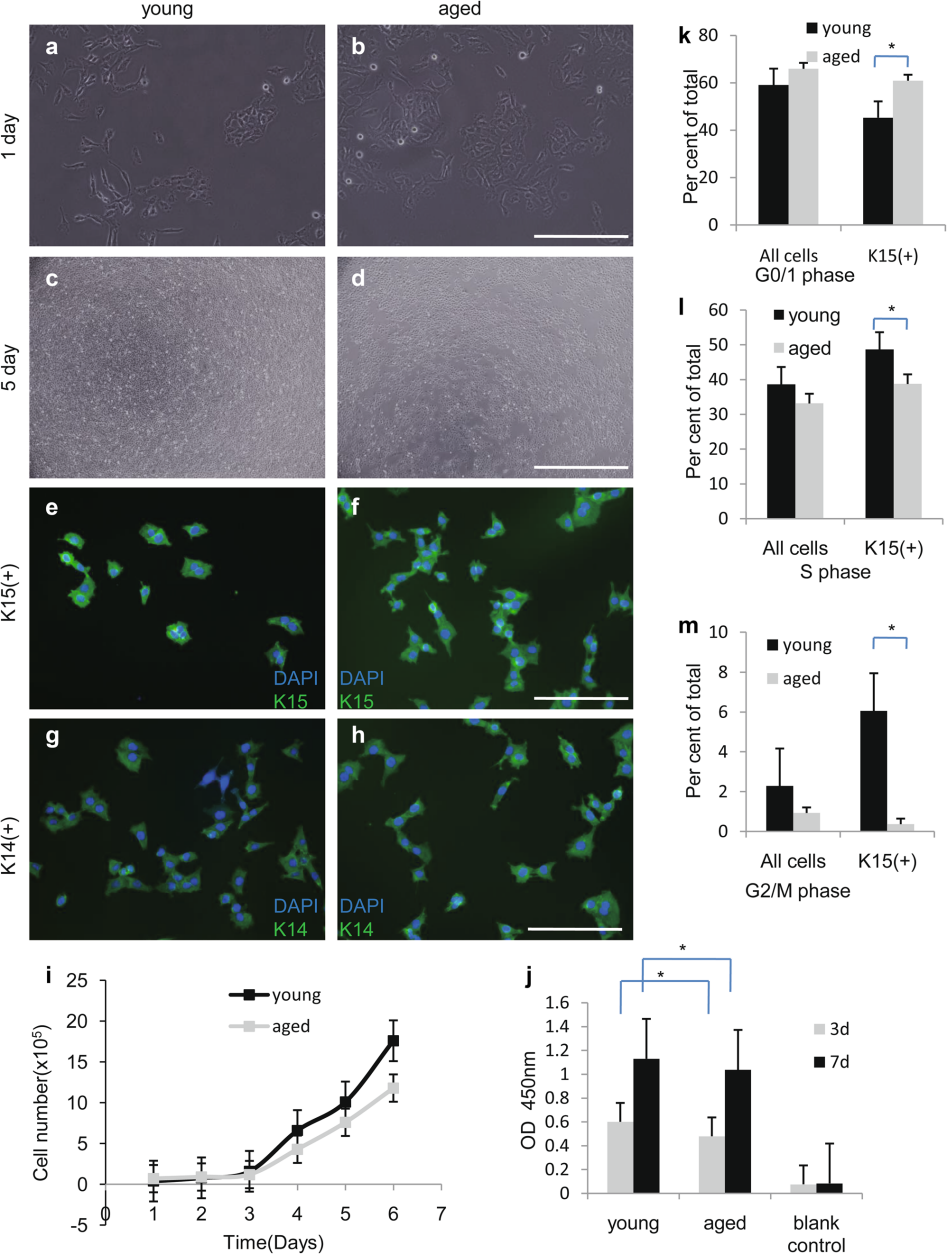
Comparison of nail stem cell proliferation and viability between the young and aged groups. **a**–**h, i** Nail stem cell characterisation. When cultured in DKSFM, the cells tended to clump together (**a, b**; scale bar = 1000 μm) and showed high proliferation capacity (**c, d, i**; scale bar = 200 μm). **e**–**h** Immunofluorescence staining for K14, K15 (green), and cell nuclei (DAPI/blue) (scale bar = 200 μm). **j** After seeding, cell viability was evaluated by CCK assays; the cell metabolic activity of the young group was significantly higher than the aged group (p = 0.04). **k**–**m** Flow cytometric analysis of total (left side graphs) or K15(+) (right side graphs) cell cycle status in young versus old nail preparations (p < 0.05). *P < 0.05. (Color figure online)

**Fig. 6:**
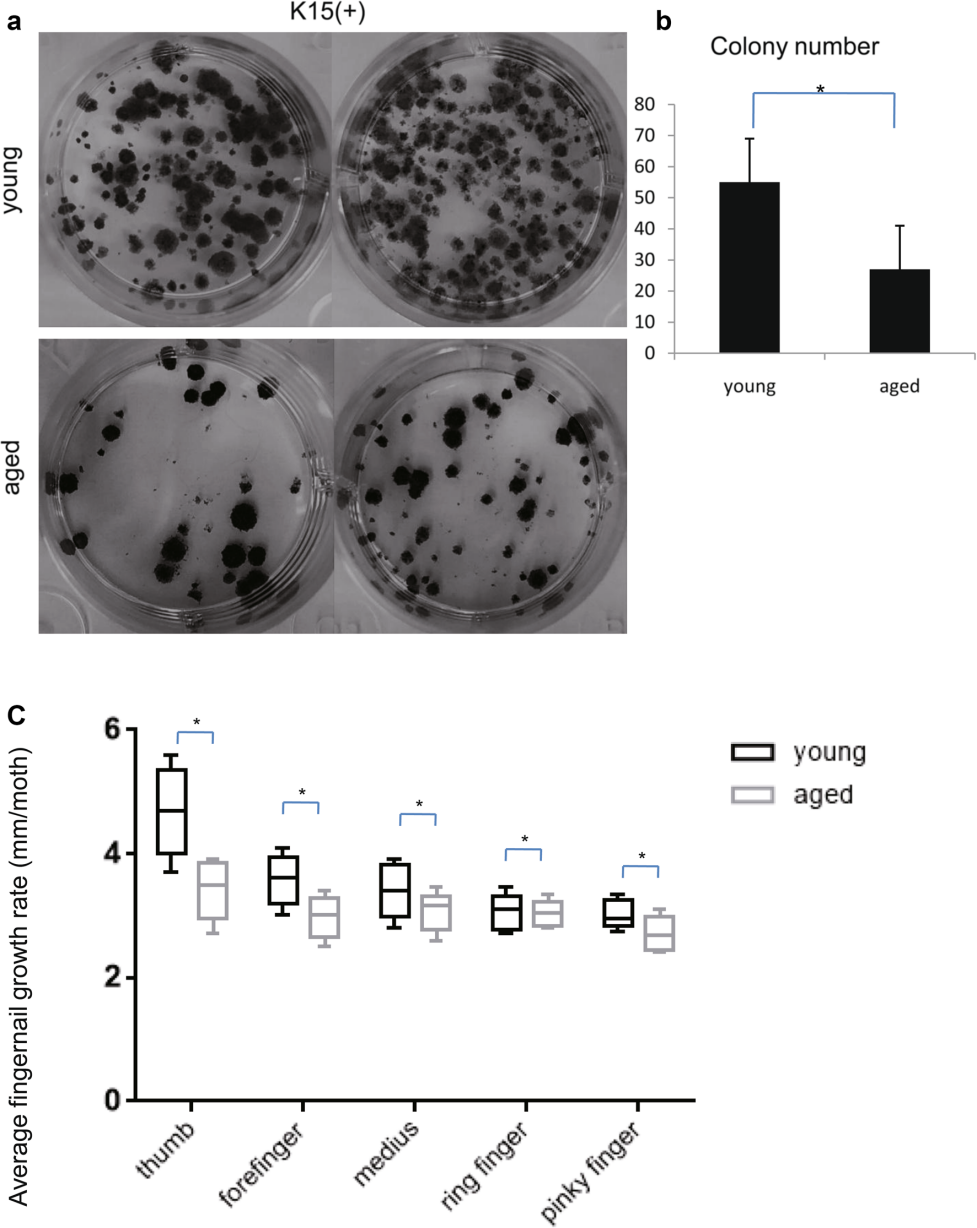
Age-associated hypofunctional in K15^+^ cells and nail growth rate. **a** Colony-forming assays of K15^+^ cells from young and aged nails. **b** Quantification of colony number (p = 0.02). **c** Comparison of average fingernail growth rates between the young and aged groups. Nails grew more slowly in the aged group compared to the young group (n = 64, p = 0.03). Average nail growth rates are presented in mm per month (defined as 30 days). Bars represent 95% confidence intervals. *P < 0.05

To determine whether the amount of those cells varies by age, we performed H&E and immunofluorescence staining for Ki67 and K15 in young and aged nail specimens (Fig. 3a–j). Through H&E staining, we discovered that the absolute number of cells, including the putative nail stem cells and other cells in the epidermis, was increased in aged samples (n = 58, p < 0.01, Fig. 3a, b, k). However, the fluorescence intensity of stem cells in aged nails was weaker than that observed in young nails. We also stained for K15 in the putative nail stem cells with different fluorescent tags (Fig. 3c–j) and the results were the same. The percentage of K15^+^ cells did not differ significantly between young and aged participants (n = 58, p = 0.5, Fig. 3l), but the gray value was higher in the young group than in the aged group (n = 58, p = 0.003, Fig. 3m). These results suggest functional differences between young and aged nail stem cells. To confirm this result, we performed immunostaining for Ki67 and K15 (Fig. 4a). Proliferation of the nail root appeared to be decreased in all aged nail samples (n = 58, p = 0.0005, Fig. 4c). Labeling for Ki67 also revealed some differences in K14^+^ cell proliferation between young and aged samples (n = 58, p = 0.002, Fig. 4b, d). By selection for nail stem cells, we were able to isolate K14^+^ and K15^+^ cells (Fig. 5e–h), which exhibited high proliferation capacity (Fig. 5a–d), but showed a difference between young and aged cells (Fig. 5i). To evaluate their proliferation capacity and metabolic activity, we performed CCK8 metabolic assays (Fig. 5j). Flow cytometry also revealed an increase in G1 phase cells concomitant with reductions in S and G2/M phase cells in aged nails (total or K15^+^ cells; Fig. 5k–m), consistent with a modest decrease in proliferation with age. In addition, we observed a striking difference in colony-forming ability of young and aged K15^+^ cells cultured in identical conditions (p = 0.02, Fig. 6a, b).

### Nails grow slower in aged versus young people

The nail plate is the product of nail matrix cells (Haneke 2015). However, it is unclear whether nail growth rate and nail stem cell proliferation are related. To determine whether nail proliferation is altered in aged people, we evaluated nail growth in 64 healthy people. Nail growth was significantly higher in the thumb compared to other digits and was slowest in the pinky finger. There were no differences in nail growth between the left and right hands. These results are consistent with previous studies (Buzalaf et al. 2006; Lavelle 1968; Orentreich et al. 1979; Yaemsiri et al. 2010). We also found that the nail growth rate in aged participants was significantly slower than in young participants, in each finger (n = 64, p = 0.03, Fig. 6c). In two cases, the nail growth rate in the right thumb was not faster than the other digits. Both of these cases were dominant in the right hand; therefore, this result may be due to wear or inhibition of growth caused by labor. The formation of nails is initiated in the area containing nail stem cells (Haneke 2015), and the nail growth rate is consistent with stem cell function based on our research. Additionally, Leung verified that NPF stem cells actively participate in nail regeneration in mice (Leung et al. 2014). Therefore, there are reasons to believe that there is a link between stem cells and nail growth, and the active proliferation of stem cells may lead to formation of the nail plate, which induces nail growth. However, further research is needed to prove this hypothesis.

## Discussion

Stem cells are defined as cells with an extensive capacity for self-renewal and the ability to generate differentiated daughter cells (Watt and Driskell 2010). Almost all stem cells have special cell markers, and the locations of stem cells are relatively constant. Researchers have previously used label-retaining cells to identify stem cells in mouse nails (Lehoczky and Tabin 2015; Leung et al. 2014; Nakamura and Ishikawa 2008; Takeo et al. 2013). Although some scholars have defined nail stem cells using embryonic nails (Sellheyer and Nelson 2012), the existence of stem cells in postnatal human nails, which are continuously growing, has not yet been determined. This is, therefore, the first article to explore human nail stem cells.

Although many previous studies have observed stem cells in mouse nails, their results differed in terms of the location of the mouse nail stem cells. Leung et al. identified nail stem cells in the NPF (Leung et al. 2014). However, some studies reported that stem cells were found in the nail matrix (Lehoczky and Tabin 2015; Nakamura and Ishikawa 2008; Takeo et al. 2013). These discrepancies may have arisen because of differences in the experimental markers, methods, and/or time periods used in each study. In view of these differences, we used the same experimental methods to test the recognised stem cell markers. We observed multi-expressing cells within the NPF and nail matrix (Fig. 2a, b). We believe that the cells we observed in human nails are the same as those cells found in mice (Lehoczky and Tabin 2015; Leung et al. 2014), which have been determined to promote digit tip regeneration.

Leung et al. demonstrated that mouse NPF stem cells express K15 and that these cells not only contribute to nail structure but also to the peri-nail epidermis (Leung et al. 2014). When nails suffer injury, nail stem cells can respond rapidly, activate, and allow the nail matrix to take part in differentiation of the nail plate during regeneration (Leung et al. 2014). K15 expression has been identified in a number of adult epidermal stem cells (Garcin et al. 2016; Leung et al. 2013; Lin et al. 2015). K14 and K19 have been confirmed to be expressed in skin stem cells (Driskell et al. 2015; Zhang et al. 2012). In our study, K14 and K19 were expressed in the same cells as K15. This reinforces the fact that the K15^+^ cells we found in human nails are likely to be one type of stem cell (i.e., nail stem cells). However, in later research, Lehoczky et al. revealed that Lgr6-expressing cells generate the nail plate. As previously characterised, Lgr6^+^ cells contribute to the growth of hair follicles and sebaceous gland structures and can differentiate into all cell lines in the skin (Snippert et al. 2010). Lgr6-GFP expression has been observed in the digit tips, particularly in the nail matrix (Lehoczky and Tabin 2015). Therefore, we chose Lgr6 as a marker for all specimen slices. We subsequently identified Lgr6 expression in K15^+^ cells. CD34 and CD29 are generally recognised as commonly occurring in skin epithelial stem cells and are widely accepted as stem cell markers (Bose et al. 2013; Hong et al. 2014; Najafzadeh et al. 2015; Ouji et al. 2015; Watt 1998; Zhu et al. 2013). Additionally, their expression has been discovered in the same cells in human nails (Fig. 2a, b). Sellheyer et al. reported that, during embryo formation, the expression of hair follicle stem cell markers was found in the NPF (Sellheyer and Nelson 2012), suggesting a nail stem cell niche in human adults that does not disappear with maturation of the embryo.

We performed statistical analysis on stem cell numbers in young and aged specimens, and found no significant difference in terms of the quantitative proportion of cells between the two groups. This trend has been confirmed in other skin stem cells, such as hair follicle stem cells (Giangreco et al. 2008; Keyes et al. 2013; Schultz and Sinclair 2016). Although some researchers detected decreased expression of mesenchymal stem cell markers in components of the human umbilical cord, as well as a trend for age-related changes of stem cell markers, we were unable to find many studies that illustrate the change of stem cell numbers with age (Alrefaei et al. 2015; Cuevas-Diaz Duran et al. 2013). Instead, regarding skin stem cells, some studies have found that the number of stem cells increases or does not vary with age (Keyes et al. 2013; Schultz and Sinclair 2016). Other stem cells have been demonstrated to increase in number but decrease in functional capacity with aging (Rossi et al. 2005), further proving that the cells we found share common features with other adult stem cells, not only skin stem cells. We simultaneously used immunohistochemistry and immunofluorescence to count the number of the putative nail stem cells, which revealed the same outcome: an increased total number of cells and an unchanged proportion of nail stem cells. However, immunofluorescence revealed another phenomenon: the fluorescence intensity decreased in the case of aged nail staining, suggesting changes with aging, which contribute to the decline of nail stem cell activity. Nail stem cells from aged nails were present in equivalent numbers in the young. Therefore, the changes in activity were not based in a decline in the nail stem cell pool. We also found that K15^+^ cells from young nails exhibited stronger proliferation ability than aged nails, suggesting that these cells retain stem-like properties during aging. In addition, the nail growth rate in aged nails was slower than that in young nails. Thus, since the nail plate is the product of nail matrix cells (Haneke 2015), the deceleration in growth rate is likely to be the result of reduced stem cell activity. This result also confirms the existence of the putative nail stem cells.

One limitation of our research is that we did not have enough samples to successfully explore the stem properties of the cells we researched, which was the major cause of insufficient evidence of nail stem cells, which will be the focus of our next work.

To our knowledge, human limbs cannot fully regenerate after amputation unless they undergo replantation, in which case the aim is to keep as much of the normal length as possible. However, when the condition of the residual limb is unsatisfactory, even replantation cannot maintain a normal length. There have been reports that fingertips could regrow in mice and children after guillotine amputation distal to the last interphalangeal joint (Borgens 1982; Douglas 1972). We found the putative nail stem cells, which express several stem cell markers, do not decrease or disappear with aging in human nails. If self-rehabilitation can be initiated by those cells, novel therapeutics could be developed for the treatment of digit defects and amputation.

## Conclusions

Our data suggest that stem cells exist in human nails. These cells express multi-stem cell markers during the whole life, but they are hypofunctional during aging.
